# Analysis of risk factors and gene mutation characteristics of different metastatic sites of lung cancer

**DOI:** 10.1002/cam4.4424

**Published:** 2021-11-19

**Authors:** Bin Wang, Shu Chen, He Xiao, Jiao Zhang, Dandan Liang, Jinlu Shan, Hua Zou

**Affiliations:** ^1^ Department of Oncology Daping Hospital Army Medical University Chongqing China; ^2^ Department of Cell Biology and Genetics Chongqing Medical University Chongqing China; ^3^ Genecast Biotechnology Co., Ltd Wuxi City China

**Keywords:** gene mutation characteristics, lung cancer, metastatic sites, risk factors

## Abstract

Risk factors vary in terms of the pattern of lung cancer metastasis and specific metastatic organs. In this study, we retrospectively analyzed the clinical risk factors of tumor metastasis in lung cancer patients and used second‐generation gene sequencing to characterize relevant gene mutations. The risk factors of different metastatic sites of real‐world lung cancer were explored to find the differentially expressed genes and risk factors in different metastatic organs, which laid a foundation for further study on the metastasis patterns and mechanisms of lung cancer. The clinical risk factors of tumor metastasis in 137 lung cancer patients who attended our department from May 2017 to March 2019 were retrospectively analyzed and grouped based on bone metastasis, brain metastasis, other distant metastasis, and no metastasis. Single‐ or multi‐factor logistic regression analysis was performed to analyze the effect of neutrophil/lymphocyte ratio/platelet/lymphocyte ratio/lymphocyte to monocyte ratio on platelets (PLTs) and bone metastasis by combining PLT values, age, pathology type, gender, and smoking history. Based on the presence or absence of bone metastasis, distal metastasis, and PLT values of lung cancer, 39 tissue specimens of primary lung cancer were taken for 773 gene grouping and gene mutation characterization. The tumor mutation load, gene copy number instability, microsatellite instability, and tumor heterogeneity among different groups were analyzed. Age and PLT level were independent risk factors for bone metastasis and distal metastasis, but not for brain metastasis. The RB1 gene was mutated during bone metastasis, and tumor heterogeneity was less in the elevated PLT group. PLT values were an independent risk factor for distant metastases from lung cancer other than the brain. Age has a significant effect on bone metastasis formation. RB1 gene mutation was significantly associated with bone metastasis.

## Graphical abstract

Age and platelet level were independent risk factors for bone metastasis and distal bone metastasis, but age and platelet level were not risk factors for brain metastasis. The RB1 gene was mutated during bone metastasis, and tumor heterogeneity was less in the elevated platelet group. Platelet values are an independent risk factor for distant metastases from lung cancer other than the brain. Age has a significant effect on bone metastasis formation. Mutations in the RB1 gene are significantly associated with bone metastasis.

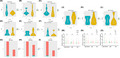

## 1 INTRODUCTION

The spread of tumor to distant organs is the cause of most cancer deaths.^1^ The mechanisms of tumor‐stromal interactions have been better understood and studied.^2^ Sequence analysis of single‐cell tumor genomes allows us to trace the clonal evolution of normal to mutated cells in primary tumors and to understand their clonal fate in multi‐metastatic polymetastases. Although there is a deep understanding of the mechanisms of tumor metastasis, limited progress has been presented in the epidemiological study of tumor metastasis.

Although the general review on metastasis cites data on the site‐specific metastasis rates, no data source is provided.^3^ The fundamental problem is that population‐based cancer registries focus on primary cancers, and few metastases are recorded. Some registries use the TNM classification, but the presence or absence of metastases at the time of diagnosis is rarely reported and there are no site‐specific data.^4^ Most of the "anecdotal" literature on metastasis rates cited are derived from clinical experience, but this literature has some limitations, such as incomplete follow‐up. However, lung cancers often metastasize to bone, brain, lung, and liver, and patients generally encounter shortened survival. Therefore, investigation of metastatic modalities is very important for patient care.

A recent study has shown that chronic inflammation may be associated with the development of lung cancer, platelets (PLTs) are not only thrombophilia effector cells but also mediate host immune responses such as innate immunity, acquired immunity and inflammatory responses.^5^ Furthermore, increasingly researchers suggest that PLTs play a role in promoting tumor cell metastasis during the development of tumors. It has been suggested that tumor cells can activate PLT through various pathways of adhesion to produce a series of bioactive molecules to regulate tumor growth and metastasis, and many tumor patients with increased PLT numbers have significantly shortened survival than those without increased PLT numbers.^6^ The association between PLT and the prognosis of lung cancer metastasis remains valuable in clinical research settings.

Lung cancer metastasis patterns and specific metastatic organs have certain patterns. Identifying metastatic risk factors for specific organs of lung cancer using multifactorial analysis has received attention, but there exist many inconsistencies in the previous reports. In this study, we evaluated the clinical risk factors for lung cancer metastasis to different organs using logistic analysis. We also explored the pattern of lung cancer metastasis and the possible molecular mechanisms using lung cancer specimens with 39 routine 773 gene sequencing, lung cancer specimens were grouped based on different metastatic organs and their risk factors, for gene mutation characterization.

## 2 METHODS

### 2.1 Patient inclusion

This study included 137 patients with lung cancer who were clinically treated at our hospital and pathologically confirmed from May 2017 to March 2019. Patients were registered for age, pathological staging, the clinical stage, gender, smoking history, blood count, and site of metastatic lesions. The normal range of PLT count was 94 × 10^9^–268 × 10^9^/L in the check system of our hospital. To deeply study the relationship between the increase of PLT count and tumor metastasis, the patients were allocated into three groups:PLT > 210 × 10^9^/L and PLT > 350 × 10^9^/L as cut‐off values. NGS with fixed panel was performed in all populations. The samples included primary lesion tissue or plasma. A total of 79 patients were tested for 773 gene sequencing and 58 patients for 1406 or 543 gene sequencing. Although several panels covered part of the same gene, the specific sites covered were not completely the same. For the consistency in sample analysis, only 39 tissue samples from primary lung cancer lesions tested for 773 gene sequencing were included in this study for subsequent mutation characterization. The study protocol and all data were approved by the Ethics Committee of Army Medical Center. All patients have signed the informed consent.

### 2.2 Data extraction and analysis

Clinical data were extracted by Army Medicine Centre and genetic testing data were sequenced and analyzed by Genecast Biotechnology Co., Ltd. After the quality test of sequence measurements, the genomic positions were first determined using the sequence comparison software bwa, with the human genome reference sequence hg19 as a template; Next, single nucleotide variation (SNVs), Indels, and copy number variation (CNVs) were detected using the widely used detection software and algorithms (such as samtools, Vardict, Freebayes, and cnvkit). The detection of SNVs, Indels, CNVs A mutation type and frequency is inferred primarily from the pileup of sequenced reads, based on the support numbers and the quality of sequencing of different bases at uniform genomic positions. All mutation types are functionally annotated using the software annovar. The original mutations were then screened for somatic SNVs according to the following screening criteria and then tumor mutation burden (TMB) and tumor heterogeneity (MATH) were calculated: (i) located in intergenic or intronic regions; (ii) synonymous SNVs; (iii) allele frequencies ≥0.002 in the database exome aggregates (ExAC) and genomes; (iv) allele frequencies of 0.05 for tumor samples and 0.01 for plasma samples was 0.01; (v) bias mutations in reads; (vi) support reads <5; (vii) depth <30.

Using the cnvkit software, the copy number values and gene values were calculated for each case as paired samples, and unstable CNI values were calculated for the whole sample based on the CNV values of the samples. If the copynumber of a gene was >4, the gene was considered to have experienced a CNV Gain. If the copynumber of a gene was <1, the gene was considered to have experienced a CNV Loss.

### 2.3 Statistical methods

Data analysis was performed using both R3.5.1 and SPSS 23.0. Based on the clinical characteristics of the patients, the Mann–Whitney *U* test was used to analyze differences in the distribution of specific blood values between the two groups. The chi‐square test or Fisher's exact test was used to analyze correlations between important clinical factors. Univariate and multivariate logistic regression analysis was performed using SPSS to identify predictors for distant metastasis. The mutated genes and tumor mutation load (TMB), MATH, microsatellite instability (MSI), copy number instability (CNI), and copy number variation load (burden of copy number) in clinical samples were analyzed using bioinformatics methods, and the Wilcoxon test was used to rank and test the mutated genes and TMB in different groups. The Wilcoxon test was used to rank and test the variance of mutated genes and TMB/MATH/MSI/CNI/Burden of copy number in different populations. Statistical analysis was performed using R software. The enrichment analyses were carried out using KOBAS 3.0 software.

## 3 RESULTS

### 3.1 Correlation of bone metastases with PLT values

**FIGURE 1:**
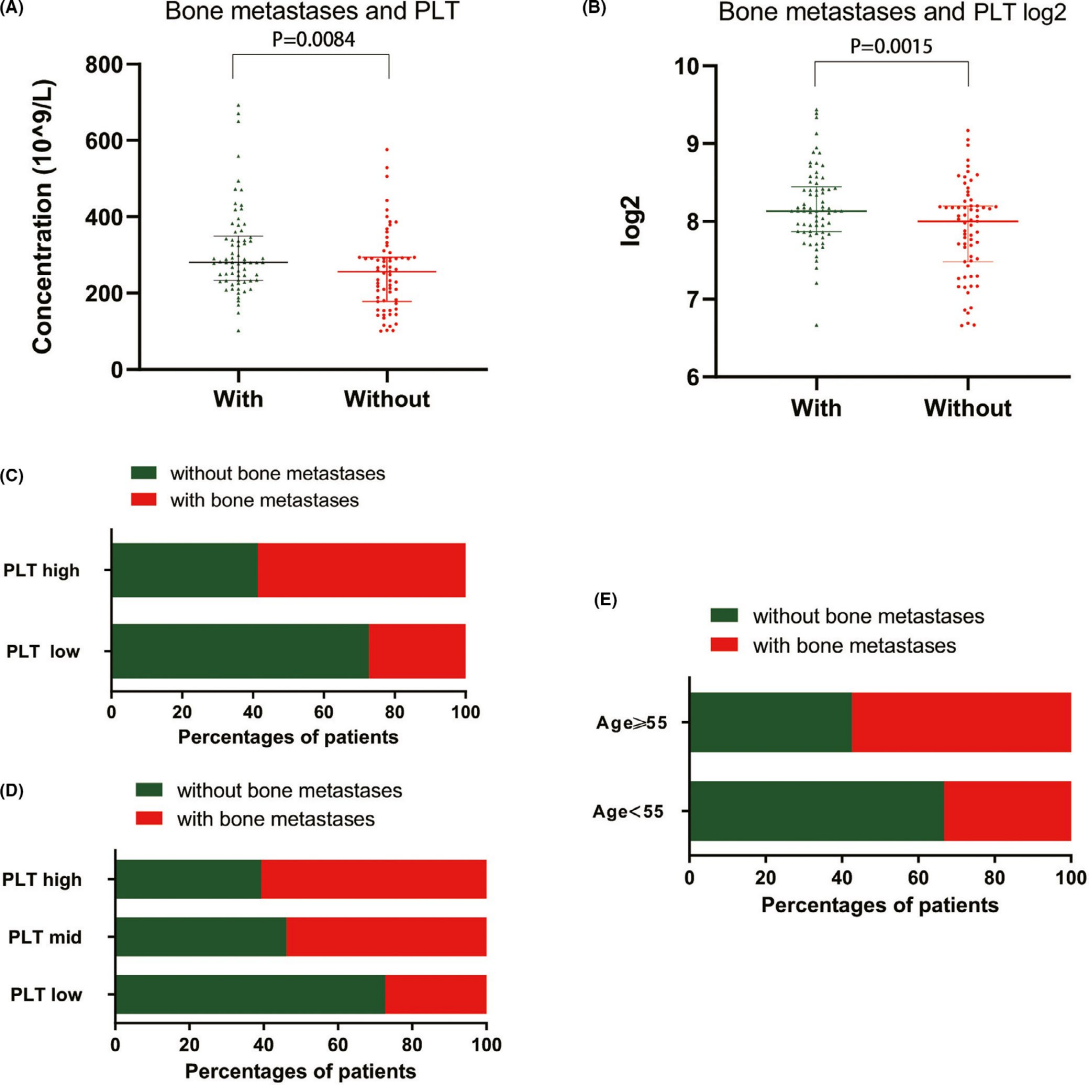
Correlation of bone metastases with multiple clinical factors. (A, B) The difference in platelet (PLT) counts between patients with and without bone metastasis (*p* = 0.0084, *p* = 0.0015). (C) Patients with PLT values >210 had a higher percentage of bone metastases (58.7%) than those with PLT values ≤210 (27.3%), with a statistically significant difference (*p* = 0.002). (D) The percentage of bone metastasis was significantly different among different PLT groups (*p* = 0.007); patients with PLT >350 (60.7%) and 210 < PLT ≤350 (57.9%) had a higher percentage of bone metastases than those with PLT ≤210 (27.3%). (E) Patients aged ≥55 years had a higher percentage of bone metastases (57.4%) than those aged <55 years (33.3%), with a statistically significant difference (*p* = 0.013)

Clinical features of the entire population were shown in Table 1. A total number of 137 patients was used to evaluate the potential association between blood cell counts and distant metastasis. Bone metastases seriously affect the prognosis and treatment of lung cancer. Therefore, a simple and accessible index was urgently needed for evaluating bone metastases. Thus, in this study, we investigated the correlation between PLT values and the risk of bone metastases. The PLT counts in patients with bone metastasis were significantly higher than those without bone metastasis (*p* = 0.0084, *p* = 0.0015) (Figure 1A,B). The percentage of bone metastases was higher in patients with PLT > 350 × 10^9^/L (60.7%) and 210 × 10^9^/L < PLT ≤ 350 × 10^9^/L (57.9%) than that in patients with PLT ≤ 210 × 10^9^/L (27.3%), showing a statistically significant difference (*p* = 0.007) (Figure 1C). Patients with PLT values > 210 × 10^9^/L developed a higher percentage of bone metastases (58.7%) than those with PLT values ≤210 × 10^9^/L (27.3%), with a statistically significant difference (*p* = 0.002) (Figure 1D). Patients aged ≥55 years had a higher percentage of bone metastases (57.4%) than those aged <55 years (33.3%) (*p* = 0.013) (Figure 1E). Moreover, univariate and multivariable logistic regression analysis showed that only age (OR = 2.588, 95% CI: 1.136–5.895, *p* = 0.024) and PLTs (OR = 3.659, 95% CI: 1.526–8.777, *p* = 0.004) were independent risk factors for bone metastasis. (Figure 1).

**TABLE 1:** Baseline characteristics of the entire cohort

	*n* (%)
Gender	
Female	57 (41.6)
Male	80 (58.4)
Age	
<55	36 (26.3)
≥55	101 (73.7)
Smoking	
No	76 (55.5)
Yes	61 (44.5)
Histology	
Adenocarcinoma	108 (78.8)
Squamous cell	19 (13.9)
Small cell	7 (5.1)
Others	3 (2.2)
T stage	
T1–2	80 (58.4)
T3–4	57 (41.6)
N stage	
N0–1	43 (31.4)
N2–3	94 (68.6)
Clinical stage	
IA–IIIA	20 (14.6)
IIIB–IV	117 (85.4)
Bone metastasis	
No	68 (49.6)
Yes	69 (50.4)
Brain metastasis	
No	93 (67.9)
Yes	44 (32.1)
Other distant metastasis	
No	107 (78.1)
Yes	30 (21.9)
Distant metastasis	
No	36 (26.3)
Yes	101 (73.7)
Platelet counts	
<210	32 (23.4)
210–350	77 (56.2)
>350	28 (20.4)

### 3.2 Correlation of distal metastasis with multiple clinical factors

**FIGURE 2:**
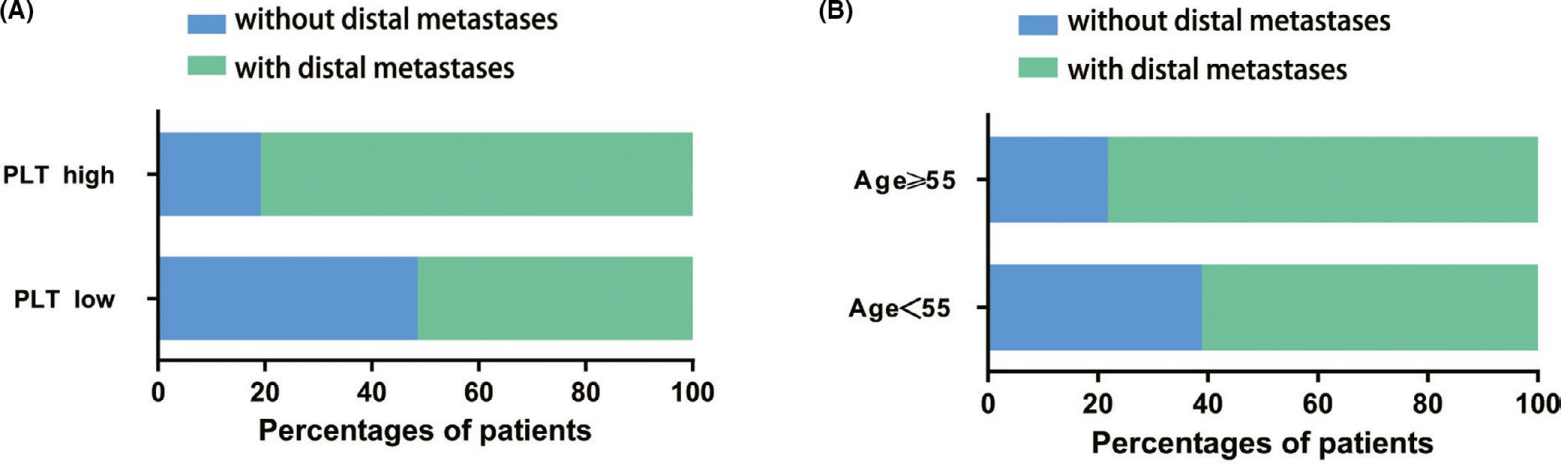
Correlation of distal metastases with multiple clinical factors. (A) Patients with PLT values > 210 developed a significantly higher percentage of distal metastasis (80.8%) than those with PLT values ≤ 210 (51.5%) (*p* = 0.001). (B) Patients aged ≥55 years had more distal metastases (78.2%) than those aged <55 years (61.1%), with a statistically significant difference (*p* = 0.045)

In addition to that, PLT values were predictive of bone metastasis, we also examined the effect of PLT values on distal metastasis. Patients with PLT values > 210 developed a significantly higher percentage of distal metastasis (80.8%) than those with PLT values ≤ 210 (51.5%) (*p* = 0.001). Patients aged ≥55 years had more distal metastases (78.2%) than those aged <55 years (61.1%), with a statistically significant difference (*p* = 0.045). However, multivariable logistic regression analysis showed that only PLT value was an independent risk factor for distal metastasis (OR = 3.808, 95% CI: 1.626–8.917, *p* = 0.002) (Figure 2).

Brain metastasis is another important metastatic mode in tumor metastasis. We performed logistic regression analysis as well as chi‐square test for clinical factors (PLT value, age, pathological staging, gender, and smoking history), respectively, of brain metastasis. The results did not reveal a statistically significant effect on brain metastasis.

### 3.3 Effect of NLR/PLR/LMR on distal metastasis

**FIGURE 3:**
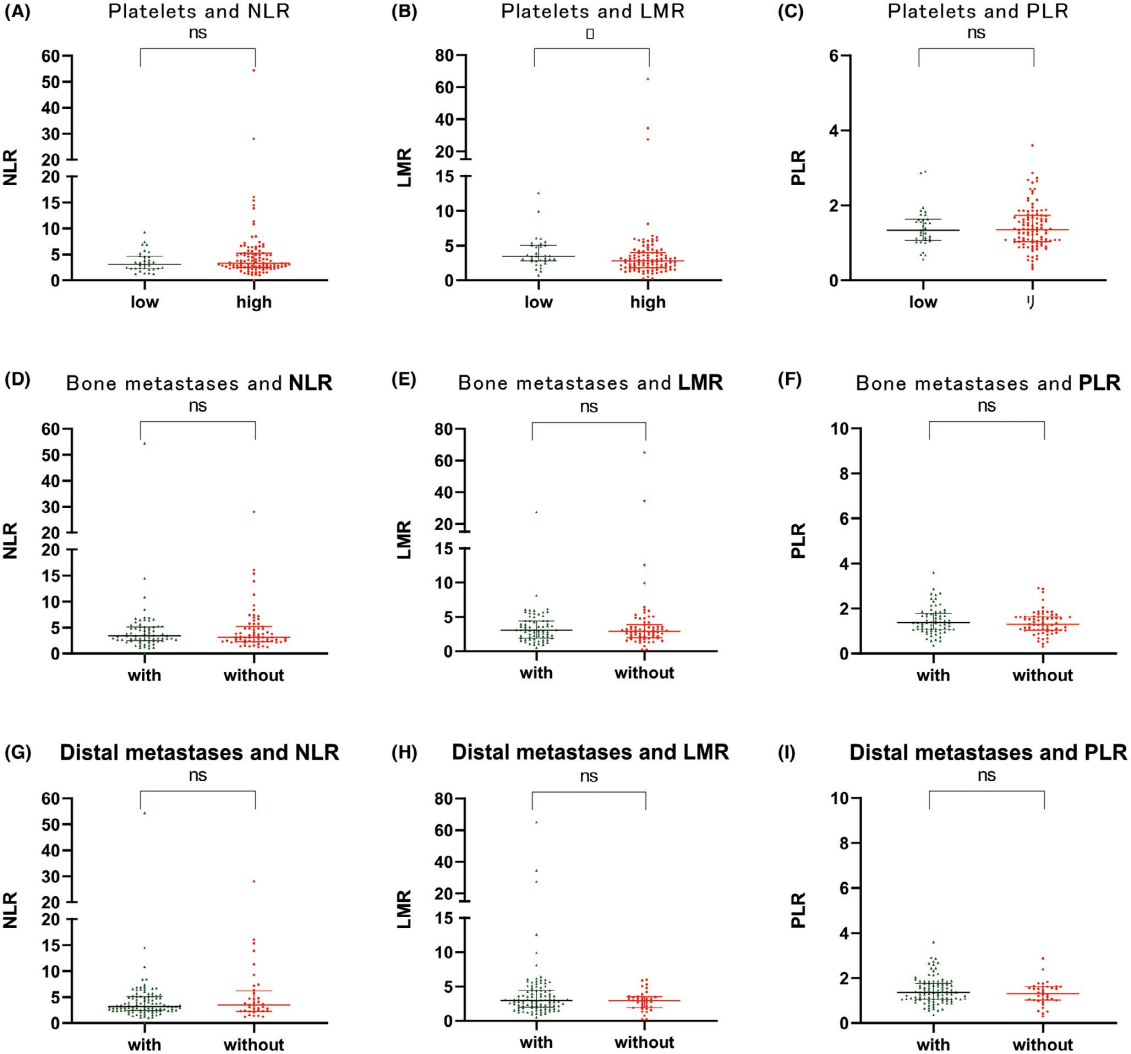
Results of the analysis of routine blood data. (A–C) There was a significant difference in lymphocyte monocyte ratio (LMR) between different platelet value groups, with a statistically significant difference (*p* = 0.029). No statistical difference in the values of NLR/PLR between low and high PLT groups was found. (D–F) No statistical difference was found between NLR/PLR/LMR and the risk of bone metastasis. (G–I) No statistical difference was found between NLR/PLR/LMR and the risk of distal metastasis

Firstly, we examined the correlation between high and low PLT count and blood count (neutrophil/lymphocyte ratio [NLR]/PLT/lymphocyte ratio [PLR]/lymphocyte to monocyte ratio [LMR]). We found the effect of LMR on PLT values had a statistically significant difference (*p* = 0.029). However, no statistical difference in the values of NLR/PLR between low and high PLT groups was found. Moreover, no statistical difference was found between NLR/PLR/LMR and the risk of bone or distant metastasis. Logistic regression analyses did not find any statistical difference between NLR/PLR/LMR values and low and high PLT groups as well as bone or distant metastasis. Taken together, these results suggested LMR could not be responsible for bone or distant metastasis (Figure 3).

### 3.4 Analysis of gene mutation characteristics among different subgroups of bone metastasis/distal metastasis/PLT high and low in lung cancer

#### 3.4.1 Characterization of gene mutations among different subgroups of PLT high and low PLT in lung cancer

**FIGURE 4:**
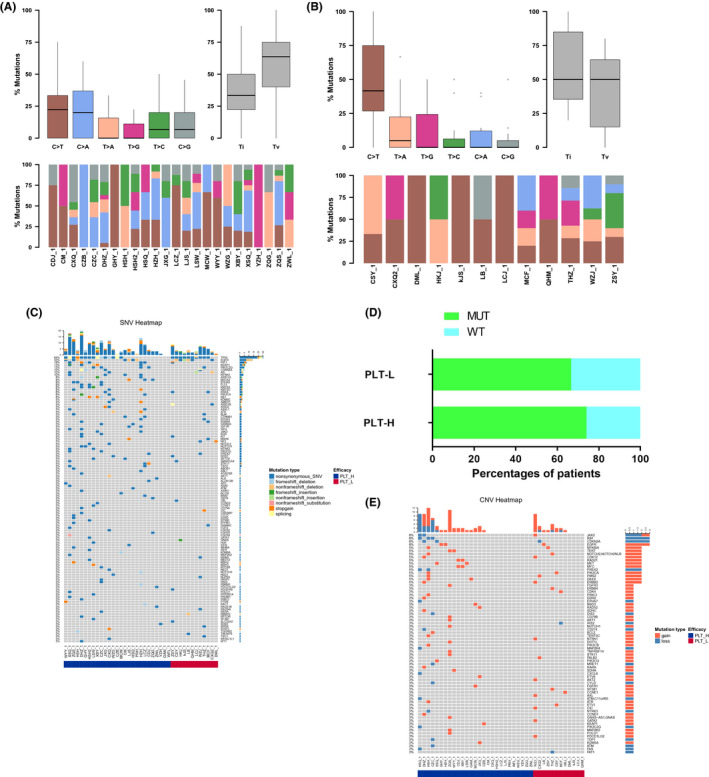
Different tumor samples may exhibit a preference for point mutation types. Statistical analysis of gene base conversion variants and reversal variants in each group of samples was performed by bioinformatics: (A) the PLT‐H group showed Tv>Ti, with the most variant types being C>A, followed by C>T; (B) the PLT‐L group showed Ti>Tv, with the most variant types being C>T, followed by T>G. (C) the PLT‐L group showed a statistically different number of mutations for ARIDIB and NF2 mutations compared to the PLT‐H group. The number of mutations in the ARIDIB and NF2 mutations was statistically different in the PLT‐L group compared to the PLT‐H group. (D) The proportional relationship between EGFR/KRAS/ALK/TP53 gene mutations and platelet high and low PLT. (E) Analysis of the difference in Somatic CNV between the PLT‐H and PLT‐low groups

The somatic SNV & Indel of the PLT‐H (PLT values > 210 × 10^9^/L) and PLT‐L (PLT values ≤210 × 10^9^/L) groups were analyzed by bioinformatics methods and statistically analyzed. The TOP10 mutated genes of the two groups were partially different, and no statistical differences were found. The gene base conversion variants and reversal variants in samples of each group were statistically analyzed using bioinformatics methods. The PLT‐H group showed Tv>Ti, where the variant type was most frequently C>A, followed by C>T; the PLT‐L group showed Ti>Tv, where the variant type was most frequently C>T, followed by T>G. We found that there was a statistical difference in the number of mutations in ARIDIB and NF2 mutations between the PLT‐H group and the PLT‐L group. (ARID1B: *p* = 0.035; NF2: *p* = 0.035). Next, we focused on the mutations in several important cancer driver genes. We found that the mutational rate of EGFR/KRAS/ALK/TP53 genes showed no statistical difference between the PLT‐L and PLT‐H groups (66.67% vs. 74.07%, *p* = 0.709). No statistical difference was seen in the analysis of SNV variant gene KEGG pathway enrichment between the PLT‐H group and the PLT‐L group. No statistical difference was seen in CNV between the PLT‐L group and the PLT‐H group (Figure 4).

#### 3.4.2 Gene mutation characteristics of different subgroups of lung cancer bone metastases

**FIGURE 5:**
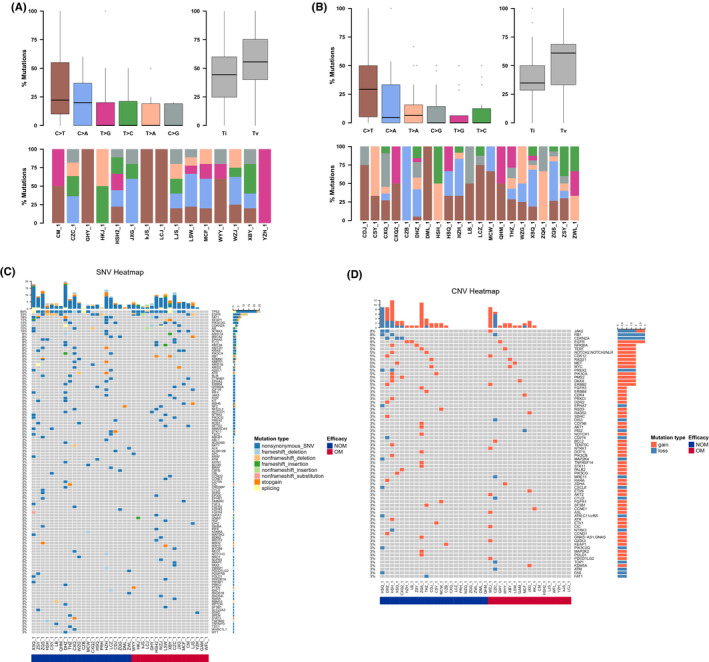
Bone metastases Somatic SNV Ti/Tv statistical analysis. Different tumor samples may exhibit a preference for point mutation type. (A) Bone metastases Somatic SNV Ti/Tv. (B) No bone metastases Somatic SNV Ti/Tv. (C) Differential analysis of bone metastases Somatic SNV. (D) Bone metastases Somatic CNV differential analysis

The somatic SNV&Indel of the two groups of bone metastases were analyzed by bioinformatics methods and statistically analyzed. The TOP10 mutated genes were partially different between the two groups, with no statistical difference. The gene base conversion variants and reversal variants were statistically analyzed by bioinformatics methods in the samples of each group. Both groups showed Tv>Ti, where the most variant types were C>T, followed by C>A. There was a statistical difference in the number of RB1 gene mutations in the bone metastasis group compared with the no bone metastasis group. Surprisingly, no statistical difference was found in the enrichment analysis of the KEGG pathway of SNV variants between the bone metastasis and boneless metastasis groups. Also, no statistical difference in CNV was observed between the bone metastasis group and the no bone metastasis group, indicating that single nucleotide variants were not the main causes for bone metastasis (Figure 5).

#### 3.4.3 Gene mutation characteristics of different subgroups of distal lung cancer metastasis

**FIGURE 6:**
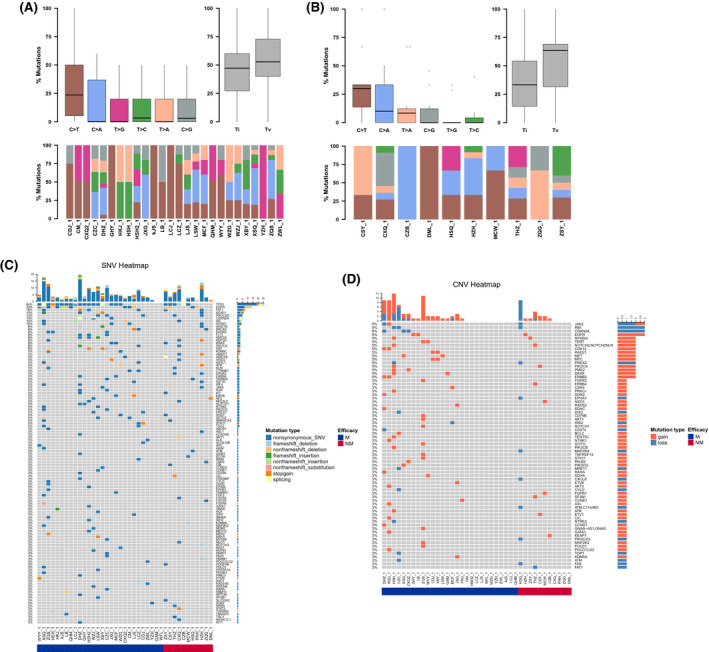
Analysis of genetic differences in different subgroups of distal metastases from lung cancer. Distal metastasis Somatic SNV Ti/Tv Statistical analysis. (A) Distal metastasis Somatic SNV Ti/Tv. (B) No‐distal metastasis Somatic SNV Ti/Tv. (C) Differential analysis of distal metastasis Somatic SNV. (D) Differential analysis of distal transfer Somatic CNV

The somatic SNV&Indel of the two groups with and without distal metastasis were analyzed by bioinformatics methods and statistically analyzed. We found that the TOP10 mutated genes in the two groups were partially different, with no statistical difference was found (Figure 6).

After that, we analyzed the point mutation types of the two groups with and without distal metastasis. By bioinformatics methods for gene base conversion variants and reversal variants in the samples of each group, we found that both groups showed Tv>Ti, with the most variant types in the group with distal transfer C>T, followed by C>A; while the variant types in the group without distal transfer C>T were similar to C>A. There was a statistical difference in the number of mutations in AMER1 and EPHA3 mutations in the group without distal metastasis compared with the group with distal metastasis. No statistical difference was found in the KEGG pathway enrichment analysis of SNV variants between the distal metastasis and no‐distal metastasis groups. No statistical difference in CNV was seen between the distal metastasis group and no‐distal metastasis group. These results suggested that single nucleotide variants were not the main causes for distal metastasis (Figure 6).

#### 3.4.4 Other genomic structural variants in different subgroups of lung cancer

**FIGURE 7:**
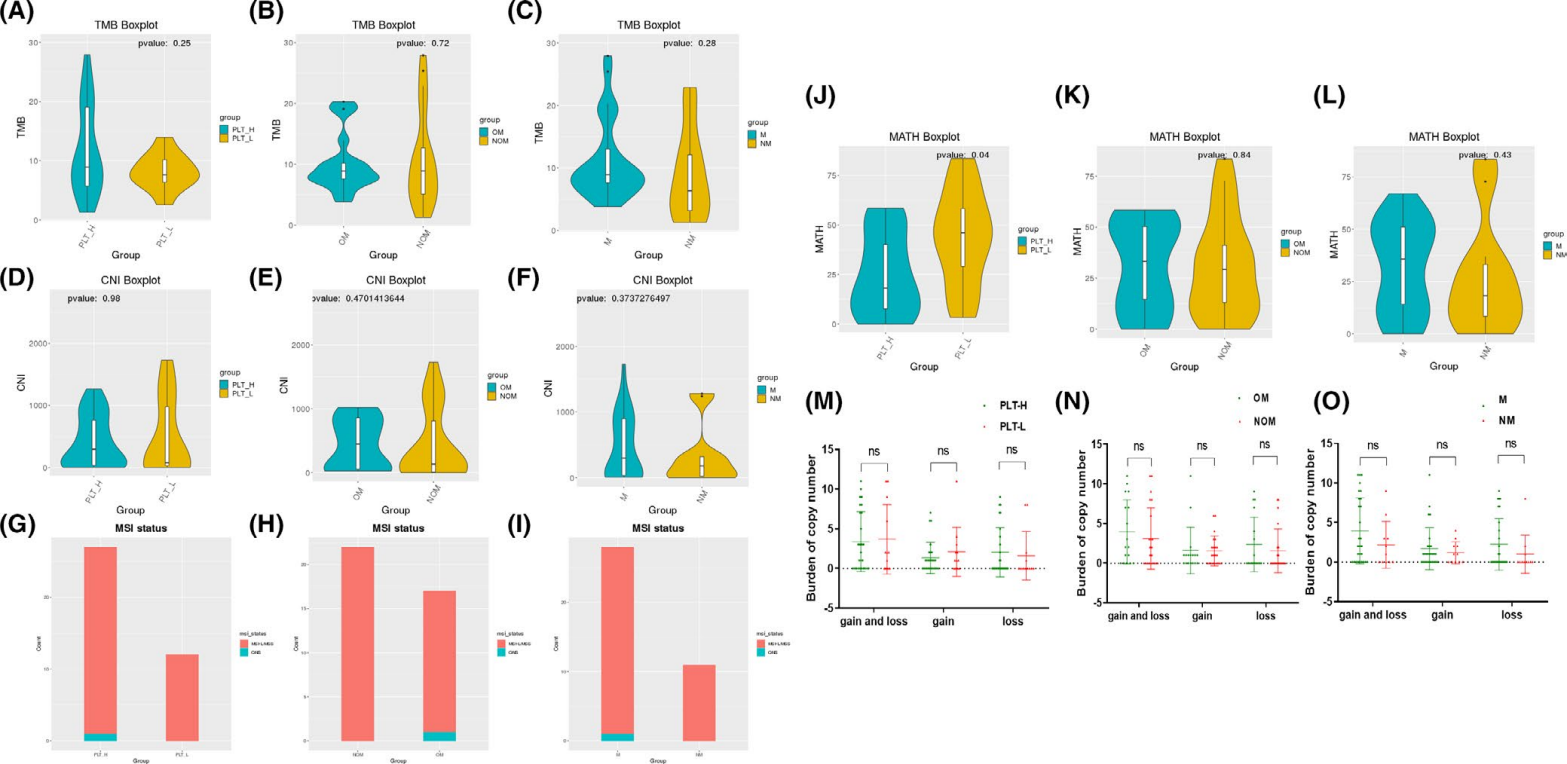
Differential analysis of other biomarkers for different subgroups of lung cancer. Tumor mutation load (TMB) differential analysis. (A) Differential analysis of platelet high and low TMB. (B) Differential analysis of TMB in bone metastasis group. (C) Differential analysis of TMB in the distal metastasis group. Copy number instability (CNI) difference analysis. (D) Differential analysis of high and low platelet CNI. (E) Differential analysis of CNI in the bone metastasis group. (F) Differential analysis of CNI in the distal metastasis group. Microsatellite instability (MSI) difference analysis. (G) Differential analysis of high and low MSI in platelets. (H) Differential analysis of MSI in the bone metastasis group. (I) Differential analysis of MSI in the distal metastasis group. (H) Differential analysis of tumor heterogeneity (MATH). (J) Differential analysis of high and low platelet MATH. (K) Differential analysis of MATH in the bone metastasis group. (L) Differential analysis of MATH in the distal metastasis group. Differential analysis of Burden of Copy Number. (M) Differential analysis of high and low platelet CN. (N) Differential analysis of CN in bone metastasis group. (O) Differential analysis of CN in distal metastasis group

Tumor mutation burden, CNI and MSI have been found to affect the degree of tumor metastasis. We found there's no statistical difference in TMB between different subgroups. No statistical differences were found in CNI between subgroups, as well as in MSI. Interestingly, there was a statistical difference in PLT high and low groups in terms of MATH (*p* = 0.04), while no statistical difference was seen in terms of MATH between the bone metastasis and distal metastasis groups. No statistical difference was seen in the burden of copy number between the different subgroups. This result suggests that single nucleotide variants were not the main causes for distal metastasis. The structural features of genes determine whether genes are prone to recombination. Hence the above data suggested that genomic structural variants were not the main influencing factors of lung cancer metastasis (Figure 7).

## 4 DISCUSSION

The relevance of thrombocytosis to tumors has been studied for a long time, with Leopold Rees first identified that thrombocytosis was associated with solid tumors more than a century ago.^7^ Current studies have suggested that PLTs are active in the whole process of tumorigenesis (including tumor growth, tumor cell extravasation, and cell metastasis.^8^, ^9^, ^10^ They also play an important role in protecting cancer cells from chemotherapy‐induced apoptosis^11^ and in maintaining the integrity of tumor vasculature.^12^ Thrombocytosis in cancer patients is associated with poor survival. Gonzalez Barcala et al. have used the increased PLT count as a risk factor in assessing the prognosis of lung cancer, they concluded that patients with high PLT had a 37% lower survival rate than those with low PLT levels.^13^ Gupta et al. have concluded that PLT‐derived lysophosphatidic acid was selected by invasive breast and ovarian cancer cells as a tumor cell pro‐divider and bone metastasis promoter of osteolysis in the process.^14^ PLT‐derived autotrophic factors and lysophosphatidic acid promote breast cancer metastasis to bone.^15^, ^16^ Moreover, PLTs direct the formation of pre‐metastatic ecotone in the bone by secreting transforming growth factor‐β and matrix metalloproteinase‐1.^17^

In the present study, PLT value was validated as the independent influence factor on bone metastasis and distal metastasis; neither age nor PLTs were influence factors of brain metastasis. It is important to investigate the mechanism of PLT involvement in tumor metastasis. Patients older than 55 years in our study had a higher incidence of bone metastasis suggested that bone age may be an important factor influencing bone metastasis in lung cancer. However, our results are contrary to the findings of Tumor Biol et al., who concluded that patients with bone metastases were younger than those without metastases,^18^ which may be due to their fewer enrolled cases. In addition, in our study, it was suggested that PLT values were an independent influence factor of distant metastases of lung cancer other than brain and brain metastasis may have different mechanisms than metastases from other sites throughout the body. Proteomic analysis confirms that cell migration‐inducing and hyaluronan‐binding protein (CEMIP) is elevated in the exosomes of brain metastatic cells but not in the lung or bone metastatic cells. The uptake of CEMIP+exosomes by brain endothelial cells and microglia induces perivascular endothelial cell branching and inflammation through the upregulation of Ptgs2, TNF and CCL/CxCL cytokines, which promote cerebral vascular remodeling and metastasis.^19^

Moreover, we screened RB1 gene mutations by characterizing different subgroups of lung cancer bone metastases. Its regulated retinoblastoma protein is a typical tumor suppressor that regulates cell cycle progression. Mutational inactivation of the RB1 gene is an oncogenic factor in various cancers, including lung cancer. Stathmin‐mediated disruption of microtubule dynamics is a key factor that induces the combined lethality of RB1‐deficient cancers and suggests that upstream factors that regulate microtubule dynamics, such as AURKA could be potential therapeutic targets for RB1‐deficient cancers.^20^ In the untreated EGFR‐mutated lung adenocarcinomas (LuADs), RB1 is excessively altered by inactivation, mainly through complex intragenic rearrangements.^21^ In the kras‐driven lung cancers, loss of RB1 promotes a glycolytic phenotype but does not alter pyruvate oxidative metabolism or glutamine inactivation.^22^ It has been suggested that EGFR/TP53/RB1 mutant lung cancers have a unique risk of histological transformation, with 25% of lung cancers exhibiting ab initio small cell lung cancer or eventual small cell transformation.^23^ Another meta‐analysis has shown that loss of RB1 function leads to a 1.6 two‐fold increase in the mortality of patients with osteosarcoma, a significant increase in osteosarcoma metastasis, and a notable decrease in osteosarcoma response to chemotherapy.^24^ Herein, more presence of RB1 gene mutations in bone metastases of lung cancer was correlated to the worse prognosis of patients, and RB1 mutations may lead to tumor differentiation and growth and promote tumor metastasis. At present, a study has revealed that the functional status of immune cell subsets may be conducive to tumor distant metastasis. Massimiliano Mazzone et al^25^ found that the presence of PoEMs in the lymphatic niche fosters lymphangiogenesis and aids cancer dissemination. Our study preliminarily found the relationship between the change of RB1 CNV and distal metastasis. The mechanism of RB1 on distant metastasis needs further clarification by studying the regulation of RB1 CNV on the immune microenvironment. However, there are only DNA detection data, no RNA sequencing, and no protein level detection, based on the current data, it is impossible to analyze the correlation between the immune cells’ activity and the distal metastases. Little is known about the association between bone metastases and RB1 gene mutations, which deserves in‐depth analysis in subsequent studies.

Searching for easily accessible prognostic indicators has been an important part for cancer research. It has been suggested that prognostic biomarkers in stage IV non‐small cell lung cancer may include NLR, LMR, PLR, and advanced lung cancer inflammatory index (ALI).^26^ This study showed that high NLR, high PLR, low LMR, and low ALI were significantly associated with poor overall survival (OS); high NLR and low ALI were significantly associated with poor OS after treatment. The present study, however, reached inconsistent conclusions, showing no statistical difference between NLR/PLR/LMR and the risk of bone metastases. It has also been reported in the literature that the mutation status of EGFR/KRAS/ALK/TP53 genes is associated with metastasis in different tissues and organs of lung cancer; in lung adenocarcinoma (LADC) patients, KRAS mutation frequency showed changes related to the metastatic site, and KRAS mutation was associated with significant poor prognosis in cases of bone metastasis.^27^ Multifactorial analysis showed that bone metastasis was a significant independent negative predictor of OS in patients with mutant and wild‐type EGFR.^28^ In this study, the presence of mutations in any of the EGFR/KRAS/ALK/TP53 genes was defined as the MUT group, and the absence of gene mutations was defined as the WT group; the mutation status of the EGFR/KRAS/ALK/TP53 genes was not found to be statistically different between the PLT‐L and PLT‐H groups. It is suggested that PLT values may not correlate with the mutation status of the above‐mentioned genes. It is derived from the regulation of other mechanisms (such as the TLR4/MyD88 pathway) to promote tumor bone and distal metastasis. Tumor heterogeneity mainly lies in gene changes. The PLT‐H group had less tumor heterogeneity in this study, which suggested that the single factor PLT involved in lung cancer metastasis was not the main reason for tumor heterogeneity.

The occurrence of SNV in the tumor eventually leads to the change of phenotype, which requires the comutation of multiple genes in the same or multiple pathways. For example, in ovarian cancer, only the simultaneous truncation mutation and missense mutation of ARID1A and PI3KCA may cause the growth of malignant metastatic tumor cells. In this study, only 37 samples were performed for NGS. It is difficult to find the difference of SNV as a binary variable between bone metastasis and no bone metastasis groups at the single‐gene level. Our research is only a preliminary exploration.

## 5 CONCLUSION

In conclusion, PLTs represent a huge biorepository of tumor‐derived and bioactive molecules. PLTs help CTC to attach to endothelial cells and provide signals to establish the pre‐metastatic microenvironment. It is important to investigate the role of PLTs in tumor metastatic spread and tumor angiogenesis. In this study, we concluded that age and PLTs were independent risk factors for bone metastasis of lung cancer, respectively, and there were more RB1 gene mutations in bone metastasis and less tumor heterogeneity in the PLT‐H group. However, because the clinical sample size in this study was small, these conclusions need to be validated by more follow‐up studies.

## CONFLICT OF INTEREST

The authors declare that they have no competing interests.

## AUTHORS' CONTRIBUTIONS

Hua Zou and Jinlu Shan designed this study. Bin Wang and He Xiao collected the samples. Jiao Zhang and Dandan Liang performed the whole‐exome sequencing of all included samples and statistical analyses. Bin Wang and Shu Chen drafted the manuscript. Hua Zou provided critical comments, suggestions, and revised the manuscript. All authors read and approved the final version of the manuscript.

## PATIENT CONSENT FOR PUBLICATION

The studies involving human participants were reviewed and approved by the Ethics Committee of Army Medical Center of PLA. Ethics Committee of Army Medical Center of PLA Approval of Medical Research Involving People Ethical Ratification No: 2020 (58). The patients/participants provided their written informed consent to participate in this study.

## Data Availability

The datasets analyzed during the current study are available from the corresponding author on reasonable request. The data of gene sequencing are openly available in GSA (Genome Sequence Archive) via the following accession: https://ngdc.cncb.ac.cn/bioproject/browse/PRJCA005887. These data underlie Figures 3, 4, 5, 6, 7 in the related article.
